# MMPI-2: Cluster Analysis of Personality Profiles in Perinatal Depression—Preliminary Evidence

**DOI:** 10.1155/2014/964210

**Published:** 2014-12-09

**Authors:** Valentina Meuti, Isabella Marini, Alessandra Grillo, Marco Lauriola, Carlo Leone, Nicoletta Giacchetti, Franca Aceti

**Affiliations:** ^1^Department of Neurology and Psychiatry, Policlinico Umberto I, Sapienza University of Rome, Viale dell'Università 30, 00185 Rome, Italy; ^2^Department of Social and Developmental Psychology, Sapienza University of Rome, Via dei Marsi 78, 00185 Rome, Italy

## Abstract

*Background*. To assess personality characteristics of women who develop perinatal depression. *Methods*. The study started with a screening of a sample of 453 women in their third trimester of pregnancy, to which was administered a survey data form, the Edinburgh Postnatal Depression Scale (EPDS) and the Minnesota Multiphasic Personality Inventory 2 (MMPI-2). A clinical group of subjects with perinatal depression (PND, 55 subjects) was selected; clinical and validity scales of MMPI-2 were used as predictors in hierarchical cluster analysis carried out. *Results*. The analysis identified three clusters of personality profile: two “clinical” clusters (1 and 3) and an “apparently common” one (cluster 2). The first cluster (39.5%) collects structures of personality with prevalent obsessive or dependent functioning tending to develop a “psychasthenic” depression; the third cluster (13.95%) includes women with prevalent borderline functioning tending to develop “dysphoric” depression; the second cluster (46.5%) shows a normal profile with a “defensive” attitude, probably due to the presence of defense mechanisms or to the fear of stigma. *Conclusion*. Characteristics of personality have a key role in clinical manifestations of perinatal depression; it is important to detect them to identify mothers at risk and to plan targeted therapeutic interventions.

## 1. Introduction

Perinatal mood disorders include a variety of clinical entities, which differ with regard to the period of onset, the severity of the illness, and psychopathological features [1, 2]. Therefore, the observation and analysis of these features have led researchers to wonder about the existence of several etiopathogenetic pathways and specific risk factors in relation to different clinical conditions [3]. In clinical practice, it is very common that the depressive symptoms seem to be an epiphenomenon of a deeper disease, which has its roots in issues concerning both the area of the integration of identity and the affective modulation. Many have written about pregnancy and maternity, focusing on the hard task that women have to deal with in this peculiar phase of their life cycle [4, 5]. Indeed, maternity is known as an interval of vulnerability, in which the physical, psychological, and relational transformations imply a deep reorganization of the inner and outer reality, a sort of “identity crisis” that allows the mother to arrange in her mind a new place to grow a new representation of the incoming baby and of herself as a parent [6]. In this transitional phase, women with a complex personality structure can develop affective symptoms. These personality structures, in which intrapsychic and relational functioning are already problematic, may become maladaptive during the delicate transition from being a daughter to being a mother.

Personality structure is considered a significant source of vulnerability for the onset, development, and treatment of various psychiatric conditions [7] and it is likely to play a role in perinatal depression. Although the relation between major depressive diseases and personality diseases has been deeply investigated, the number of works concerning personality organization as a factor of vulnerability in perinatal depression has been limited in the psychiatric literature [8, 9]. The studies conducted so far suggest that women with obsessive-compulsive, avoidant, or dependent personality disorders have greater risks of developing a major depressive episode during pregnancy [10]. This is also the case for women with borderline personality organization [11], who may have specific difficulties in dealing with developmental challenges typical of the transition to parenthood.

A specific bond between obsessive-compulsive/dependent personality traits and depressive perinatal risk can be found among the features related to these profiles. They are often associated with the tendency to experience feelings of guilt and inadequacy, low self-esteem, and lack of autonomy, along with brooding, lack of assertiveness, and hypersensitivity toward refusal. The tendency of these mothers to experience a state of growing anxiety, along with a predisposition to face intimate relationships with an attitude of hyperresponsibility, can transform the engagement in child care into an emotional burden that can overwhelm these patients, leading to a depressive condition. Akman et al. noticed that women with an avoidant, dependent, or obsessive-compulsive personality disease had greater chances of postpartum depressive onset [10]; Uguz et al. highlighted that the most significant signs during the first year after giving birth were higher scores on the Edinburgh Postnatal Depression Scale (EPDS) and the existence of a personality disorder, particularly obsessive-compulsive and dependent disorders [12]. Further research linked the fear of relationships (typical of a C-cluster personality disorder) with postnatal depression symptoms [13]. Dennis et al. underlined how this peculiar clinical condition might be connected to low self-esteem, poor coping, and high personal perception of stress [14].

Another academic work has focused on the relation between borderline personality disorder (BDP) and perinatal depression, pointing out that these women's general ways of behaving might interfere with the reorganization of their identity and of their relationships during the transition to motherhood [15]. Newman and Stevenson highlighted that mothers with BPD had difficulties in deciphering the emotional states of the child, as they were affected by their continuous shifting among experiences of hostility, helplessness, excitement, or dissociative states, which totally undermine the sense of continuity of their Self. These BPD-affected new mothers perceive themselves as incompetent parents, experiencing feelings of unfamiliarity, oppression, and anger, declaring themselves less satisfied with their maternity experience, compared to other mothers [16].

According to Bland et al., BPD-affected women fail to respond adequately to the baby's needs and to read his states of mind correctly [17]. The main outcome is a higher risk that their children might experience emotional neglect or abuse [18]. Two studies found that BPD-affected mothers are less sensitive and less able to help babies in organizing their activities [19, 20]. As a consequence, the baby appears less prompt in answering his mother and less interested in relating to her [11]. Mothers with BPD are more often frightened by their child, as the child appears frightened in front of them [21]. Mothers with BPD also report higher levels of stress as a result of parental responsibilities [22], often becoming authoritarian. Over the years their children tend to have oppositional behavior [18]. Using the Five-Factor Model, other clinical trials showed different empirical evidence that certain personality characteristics, such as neuroticism, are considered vulnerability factors for perinatal depression [23–26]. Using the Temperament and Character Inventory (TCI), Joseffson and Sydsjö showed that women with postpartum depression have significantly higher scores than the control sample in respect to the harm avoidance (HA) and lower scores in the self-directedness dimensions (SD) [27].

Although the Minnesota Multiphasic Personality Inventory-2 (MMPI-2) is one of the most used tests in the study of personality disorders, in literature a few studies have used MMPI-2 in samples of women with perinatal depression with the specific aim of identifying the personality profiles underlying a depressive state. Elisei et al. administered a battery of tests including MMPI-2 and analyzed the epidemiology, risk, and protective factors of perinatal depression. However, the study of personality was not taken into consideration and no results about this specific topic were reported [28]. Robertson identified different personality structures outlined by MMPI-2 but in a specific target population of addicted patients in an outpatient program for treatment of addiction in the perinatal period [29]. The present study is the first in which a general population of women with perinatal depression was investigated using MMPI-2 in an Italian sample, in order to clarify the personality structure underlying the mood disorder.

We propose that the study of personality traits underlying the depressive symptoms arising in the perinatal period is fundamental, because they represent the vulnerability mechanism with which the pathology itself is established, determining the clinical presentation, course, and response to treatment. The evaluation of the personality structures could lead to establishing “risk profiles” that may become a target for primary and secondary prevention. Therefore, our study aims to assess personality characteristics of women who develop perinatal depression, providing Italian data for the first time.

## 2. Material and Methods

### 2.1. Study Population and Sample Selection

The study was conducted using data collected during a screening carried out by the “Perinatal Disorder Unit,” affiliated with the Department of Psychiatry of Policlinico Umberto I in Rome. A sample of 453 women in the third trimester of pregnancy, aged between 18 and 45 years, was considered [30]. The exclusion criteria were the refusal to provide informed consent, age under 18 years, the diagnosis of mental retardation or schizophrenia, and poor knowledge of Italian or other verbal communication limitations that compromised the ability of the subject to follow the research protocol. Before being enrolled in the study, participants were informed of the nature and objectives of the research. Enrollment was voluntary and both verbal and written consent were obtained. The study was approved by the local ethics committee and was therefore conducted in accordance with the ethical standards laid out in the 1964 Declaration of Helsinki and its later amendments.

Ninety-two positive women at screening (scoring ≥ 12 at EPDS) were contacted by phone and invited to participate in a clinical interview carried out within the first month after delivery by a specialized psychiatrist team at the “Perinatal Disorder Unit.” DSM-IV was used as the diagnostic standard. We confirmed the diagnosis of postpartum depression based on the Structured Clinical Interview for DSM-IV Axis I Disorders (SCID-I) [31]. However, current DSM-5 criteria reclassify postpartum depression in a broader perinatal depression category, which we refer to throughout the paper. The diagnosis was confirmed for 55 women who completed the research protocol and were therefore included in the study group.

### 2.2. Questionnaire

All patients included in the study group were invited to perform a clinical interview with a specialist psychiatrist and completed the following questionnaires:

(i) a survey data form: a self-administered questionnaire to collect information on sociodemographic aspects, details of pregnancy, family, and personal psychiatric history, life stressors, and familiar or marital conflicts;

(ii) the Edinburgh Postnatal Depression Scale (EPDS): a 10-question self-rating scale specifically designed for women who are pregnant or have just had a baby. This scale has been proven to be an efficient and effective way of identifying patients at risk for perinatal depression. A score equal to or greater than 12 indicates moderate to severe depression. The questionnaire was validated in an Italian version and has a high level of validity, reliability, and internal consistency [32];

(iii) the Minnesota Multiphasic Personality Inventory-2 (MMPI-2): a standardized psychometric test of adult personality and psychopathology. MMPI-2 consists of 567 items. The most typical use of MMPI-2 is to evaluate the overall profile configuration of the 10 clinical scales, particularly the combination of the two or three scales with the highest scores, which is called the code type. Three validity scales are also part of a standard interpretive procedure of MMPI-2. Thus, 10 clinical scales and three validity scales are included in graphical presentations of the MMPI profiles. *T*-scores are used in MMPI-2 evaluation (standardized scores in which the scores of the original norm group indicating normality are set to 50 on each of the MMPI-2 scales). The clinical scales in MMPI-2 are scale 1 (Hs, hypochondria), scale 2 (D, depression), scale 3 (Hy, hysteria), scale 4 (Pd, psychopathic deviate), scale 5 (Mf, masculine-feminine interests), scale 6 (Pa, paranoia), scale 7 (Pt, psychasthenia), scale 8 (Sc, schizophrenia), scale 9 (Ma, mania), and scale 10 (Si, social introversion/extroversion). The main validity scales are as follows: L (lie scale), F (infrequency), and K (correction scale). MMPI-2 operates with a *t*-value of ≥ 65 indicating an elevated score. This cut-off score indicates distinct psychological problems or pathology. MMPI-2 contains several new clinical scales in addition to the original 10 main scales. Some of the most important ones are the 15 scales created by a procedure that combines rational and statistical methods called content scales. According to Butcher, the content scales are believed to have higher face validity and to reflect more homogenous clinical concepts than many of the predecessors in MMPI. Both the traditional clinical scales and the new content scales are used in the present study. MMPI-2 has been translated into Italian and it has been thoroughly tested and adapted [33, 34].

### 2.3. Statistical Analysis

MMPI-2 clinical and validity scales were used as predictors of group membership in a hierarchical cluster analysis. Since the goal was to cluster cases, all scale scores were standardized to reduce potential biases due to differences in the variance of each variable. Ward's method was used with squared Euclidean distance as a proximity measure. Compared to other classification algorithms, Ward's method is deemed the most appropriate for quantitative variables, as it takes an analysis-of-variance approach to join clusters (i.e., minimizing within-cluster variability). Furthermore, it generates highly homogeneous clusters compared to alternative methods [35]. The clustering process starts with as many clusters as the clinical cases in the dataset (i.e., each case is a cluster itself). The first two clusters that are merged together are those having the lowest squared Euclidean distance (indicating the most similar profile across the MMPI-2 scales). Then, the distance between clusters is recalculated and clusters with the lowest distance are subsequently merged. The process carries on iteratively as long as all cases are merged into a single general cluster by sequential agglomeration steps. The distance at which clusters are merged together at each step is called fusion distance.

Although there is no “gold standard” for determining the “right” number of clusters in a dataset, an inconsistent increase in the fusion distance provides evidence that the clusters joined at this stage are relatively far. As a result, it is deemed appropriate to stop clustering at one prior stage. To detect such inconsistent increase, we inspected the scree plot, which is a graphical representation of the fusion distances at each agglomeration step. Since cluster solutions are deemed not perfectly replicable across clustering algorithms and proximity measure used, cross-validating clusters by a *k*-means algorithm is recommended [36]. Unlike the hierarchical method, the *k*-means algorithm required a priori specification of the number of clusters, which is based on the aforementioned hierarchical analysis, in our particular case. In order to interpret the clusters, a descriptive analysis of the MMPI-2 profile was carried out.

## 3. Results

### 3.1. Characteristics of the Study Population

The sample's mean age varied from 20 to 45 years (M = 35.10; SD 5.54). The mean score on EPDS was 18.49 (SD = 4.88).

As shown in Table 1, the majority were married or with a stable partner. Additionally, they had high educational levels and most of the participants were employed. Most women were pregnant for the first time, while a minority reported complications during pregnancy or had suffered from a medical condition.

Most women reported a positive personal history of psychiatric disorders and had a family history.

With regard to stressors that had occurred in the last year, half of the women reported having suffered mourning, about a half reported illness of a relative, and a minority reported job loss or economic difficulties.

### 3.2. Cluster Analysis

**Figure 1:**
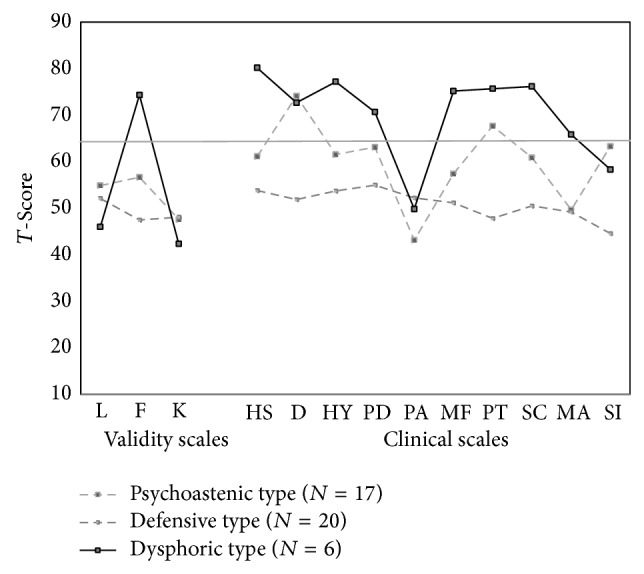
MMPI-2 cluster profiles.

In order to determine an appropriate number of clusters to be cross-validated by *k*-means clustering, we carried out a hierarchical analysis and inspected the scree plot. As a result, we detected three major discontinuities in the fusion distance, suggesting that a three-cluster solution could be a compromise between cluster generality and cluster specificity. The analysis yielded two major clusters comprising 26 and 20 cases, respectively. Besides that, a third cluster also emerged comprising 9 cases. Next, we switched to *k*-means clustering with prior specification of three means as group centroids. The *k*-means clustering yielded clusters of approximately the same size as the hierarchical clustering method. In order to classify cases in a reliable way, we combined the two different case classifications in a 3 × 3 contingency table. The analysis yielded a statistically significant association (*χ* ^2^ = 78.86; df = 4; *P* < .0001). Specifically, the clusters resulting from hierarchical analysis were replicated by *k*-means with 74%, 87%, and 67% agreement rates for clusters 1, 2, and 3, respectively. In order to arrive at a meaningful interpretation of clusters, we compared the profile of each cluster on the MMPI-2 scales. Only cases classified the same by each clustering method were considered for interpretation.  Figure 1 reports clusters' profiles.

As shown in Figure 1, women with perinatal depression revealed an elevated personality profile in most cases, indicating severe psychological disturbance, even if we found a cluster with an “apparently common” profile. The profile configurations for the two clinical groups are similar for some characteristics. In both samples the similarity was the elevation of scales 2 and 7 and the presence of a specific configuration of the clinical scales 4 and 6, even with different elevations. The 4-6* configuration* is commonly called “*characterial V*” [37] and it is generally associated with emotional distress characterized by brooding, dysphoria, and anhedonia. A subject with this profile is generally stubborn, argumentative, and angry. She externalizes blame for her anger and is not usually able to control its expression. She broods and worries constantly over what is happening to her; she is suspicious, very sensitive to criticism, and resentful of any demands being placed on her. Her solution for behavioral change is to have others change to meet her expectations. She is likely to have a history of poor interpersonal relations and often sees her family as extremely uncaring.

A cluster analysis for the whole sample identified three clusters of personality profiles. The selection criteria consisted of the 10 clinical scales in MMPI-2. The sample of women with perinatal depression was divided into subgroups based on these three clusters (Table 2).

The* first cluster* comprised 39.5% of women with perinatal depression. Women in cluster 1 showed an elevated personality profile in which clinical scales 2 (*T*-score = 74.12) and 7 (*T*-score = 67.65) were elevated, while the others were not elevated beyond the normal range. The MMPI-2 configuration for this group was less elevated than the other clinical group and the respondents in this cluster first of all portrayed an elevated *2*-*7 code type*, leading us to label this cluster* “the psychasthenic type.”* Women with this code type are characterized by chronic, deeply ingrained depressive features in conjunction with extensive feelings of inadequacy and guilt.

“They lack self-confidence and feel insecure, inadequate and inferior. Their major symptoms include depression, nervousness and obsessions. They are ruminatively introspective and show excessive indecision, doubts and worry. They are obsessed with their perceived personal deficiencies and view themselves as useless and no good at all. Their lack of drive reflects their depressive cognition and negative expectations. They are likely to overreact to minor stress with agitation, guilt and self-punishment. They are self-deprecating and try to make others feel superior by focusing on their weaknesses and inadequacies. They refuse to recognize their extensive dependency on others. They are passive and dependent in her relationship with others and often report marital problems” [38].

The defense mechanisms are in the normal range. The indicators of the difficulty of treatment also appear normal, indicating a positive compliance with psychological/psychotherapeutic intervention. Even if it is the most elevated configuration, it is possible to underline the presence of the 4-6 configuration in a subclinical range.

*Cluster 2* consisted of patients with a more “normal” personality profile, comprising 46.5% of the sample. This group had *T*-scores of <55 on all ten clinical scales. However, interestingly, the control scale portrayed the typical V configuration, with L and K scales higher than F. This configuration is usually associated with people who try to avoid or deny unacceptable feelings, impulses, and problems and try to present themselves in the best way possible. Also these people tend to have a simplistic view of the world in terms of good or bad and should have adequate social-adjustment disorders or in the worst cases, light behavioral disorders. We labeled this group* “the defensive type”* as they did not portray any psychological disturbance but seemed to have adopted a defensive disposition in answering questions.

*Cluster 3* comprised 13.95% of the patients. A feature common to all members of this group was their extremely elevated personality profiles, in which eight of the clinical scales had a *T*-score above 65. As shown in Table 2, in addition to the previously mentioned 4-6 configuration, scales 1, 2, 3, 7, and 8 were highly elevated, with different configurations: the 1-3/3-1 and 7-8 configurations.

The* 1*-*3/3*-*1 code type* is commonly called the* “conversion V”* [37]. In this code type, depressive elements are associated with the elevation of scale 1 (Hs scale, an indicator of somatic anxiety and hypochondriac symptoms) and scale 3 (Hy scale, an indicator of somatic symptoms associated with conversion mechanisms and affective denial, including psychological manipulation of interpersonal relationships, emotional insecurity, and need for confirmation). In this cluster, the anxious elements are more relevant than the obsessive ones and they seem to be expressed through the body: women often report uncertainty toward their body's modification and feeling inadequate with the new physical situation. Often they report concerns, fears, and panic states that seem to be linked to the dynamics of the “loss” and “lack” of body identity.

In this group we also found* elevation in scales 7-8*, which indicates instability, a tendency toward odd thinking, and social alienation. This was associated with the depressive elements previously recognized by the elevation of scale 2. Finally, in this cluster the *4*-*6 configuration* becomes particularly important, outlining the presence of dysphoric mood linked to characteriological elements associated with the neurotic (1-2-3) and psychotic (6-7-8) triads. Specifically, in this configuration, scale 4 (Pd, psychopathic deviate) is a good indicator of aggressive tendencies related to intolerance of social norms, family conflict, and social alienation. Scale 6 (Pa, paranoia) is a good indicator of distrust, susceptibility, interpersonal sensitivity, and a tendency toward persecutory ideation in response to stressful situations. This configuration is usually detectable in female and is associated with anger, hostility, inability to directly express these feelings (passive-aggressive style), and dysphoric mood. These are often associated with mistrust and suspiciousness. These people often complain substantially and are dependent on and overly needy for attention and affection. They often report sexual problems, as well as family and marital conflicts. Compliance with psychological/psychotherapeutic intervention seems to be poor, while defense mechanisms and the ability to manage emotional distress are found to be below average in almost all cases. This profile is often found in subjects with BPD or, in any case, with a cluster B personality disorder, so we labeled this cluster* “the dysphoric type.”* In our sample, the association of these elements with the elevation of scales 2 and 7 could exclude the possibility of acting out of the aggressive components.

Table 3 gives an overview of the results of these three clusters on the MMPI-2 content scales. The MMPI-2 content scales were less elevated compared to the scores on the main clinical scales. The “psychasthenic type” had elevated scores on generalized anxiety/negative affectivity, depression, and work problems. These figures are shared even by the “dysphoric type,” which also presents elevated scores for health concerns, bizarre mentation, and anger. The “defense type” did not have elevated scores on any of these 15 subscales. However, the content scales confirmed the impression from the main clinical scales. The “dysphoric type” had the most elevated scores of the three groups on eleven out of fifteen subscales, whereas the least atypical profile configuration was revealed among the “defensive type.” The group differences were significant in eleven out of fifteen comparisons according to univariate analysis of variance and were revealed for the scales for anxiety, fears, obsessiveness, depression, health concern, bizarre mentation, anger, low self-esteem, social discomfort, work interference, and negative treatment indicators.

In analyzing the frequency distribution of the variables shown in Table 1, there were no significant differences between the three clusters. Instead, there was a statistically significant difference between the average scores on EPDS in the three clusters (Table 4).

### 3.3. Discussion

The hypothesis of our study was that the personality traits underlying the depressive symptoms arising in the perinatal period are fundamental, because they represent the vulnerability mechanism with which the pathology itself is established, thus determining the clinical presentation, course, and response to treatment. So, the aim of our study was to assess personality characteristics of women who develop perinatal depression.

Although MMPI-2 is one of the most widely used tests in the study of personality disorders, it had not been used in the study of women with perinatal depression with the specific aim of identifying personality profiles that could underline a depressive state. This was the first study in which women with perinatal depression were investigated with MMPI-2 in an Italian study population. We used cluster analysis, a statistical technique, to identify different personality profiles in the sample based on the results obtained from MMPI-2.

This study demonstrated that women with perinatal depression are not a homogeneous group and that they may be divided into 3 different clusters based on personality configuration: 2 clinical clusters (cluster 1: psychasthenic type and cluster 3: dysphoric type) and a seemingly normal one (cluster 2).

Both clinical clusters typically portray a 4-6 configuration, which is generally associated with emotional distress, characterized by brooding, dysphoria and anhedonia, and difficulty in controlling the expression of anger, blame for their anger, sensitiveness to criticism, and resentfulness of any demands being placed on them.

The two different clinical clusters even present specific characteristics that are described as follows.

(i) Cluster 1 (*2*-*7 code type*), labeled* “the psychasthenic type,”* describes characteristic depressive profiles associated with elements of anxiety, inhibition, and phobic or obsessive components. This group had a much less elevated profile than the members of cluster 3. The profiles are often reactive in nature, with validity scales and defensive elements (which indirectly indicate the coping skills) in the normal range. The most characteristic features are pessimism, lack of motivation and energy to deal with current problems, loss of interest and pleasure in usual activities, poor self-confidence, brooding, and distrust of their reaction capability. They are associated with feelings of being overwhelmed by the current issues, difficulty to project the future.

This group of women showed a tendency to experience discomfort with respect to modification and relational identity that occur during the transition to parenthood, experiencing feelings of inadequacy and low self-esteem. These are associated with a low sense of autonomy, fatigue, and a tendency to put in place mechanisms of rumination at the level of thought to counter the belief of “not being able” to face a new situation. These characteristics, together with a hypersensitivity to rejection and the tendency to experience intimate relationships with an attitude of hyperemotional responsibility, can transform the engagement in child care into an emotional burden that can overwhelm patients with feelings of guilt and inadequacy. These feelings become the basis for recurring concerns (particularly about the mental and physical health of the child) which could eventually lead to slipping into depressive conditions characterized by specific psychasthenic features.

The features presented by this group of women, such as fixation on organization, interpersonal sensitivity, and obsessionality, may overlap with some traits exhibited by personality structure called* typus melancholicus*, which is the most important personality structure involved in the development of major depression, in European psychopathology. Previous studies have suggested that this kind of personality is at great risk of developing perinatal depression because of the incapacity to creatively manage situations of role conflict (like motherhood). Indeed, these women cannot avoid behaving with feverish perfectionism. They develop an exaggerated preoccupation towards the unborn child and hostility towards people and events that are experienced as obstacle to their search for perfection. They do all they can in planning how not to neglect their duties as a mother, wife, and worker. These duties cannot be delegated to others without experiencing guilt or feelings of being a “bad mother,” paving the way for falling into a depressive condition [39].

(ii) Cluster 3 (*1*-*3/3*-*1 code type*), labeled “*the dysphoric type,*” describes profiles characterized by major depressive elements associated with functional somatic symptoms, anxiety expressed through the body, and uncertainty for body's modification linked to the dynamics of the loss and lack of its physical identity. In our sample, this code type was often associated with the 4-6 configuration (characterial V) which presents characteristics of anger, hostility, passive-aggressive style, and dysphoric mood, often in association with mistrust, suspiciousness, interpersonal sensitivity, and a tendency toward persecutory ideation in response to stressful situations. This profile is linked to a tendency to establish conflictual relationships, either in the family or in other social interactions, and to use of primitive defense mechanisms such as projection in stressful situations.

This group displayed an MMPI-2 profile that was decidedly more elevated than the previous one, indicating a higher level of psychopathology.

This profile is often found in subjects with BPD or a cluster B personality disorder. The borderline personality organizations specifically interfere with the reorganization of identity and relational requirements in pregnancy and motherhood. These mothers experience difficulties in understanding the child's emotional states and have oscillations between states of hostility, anger, helplessness, and dissociative withdrawal which radically put into question the sense of continuity of the Self. This group of women manages discomfort resulting from “identity transformation” initially through brooding, as in the previous group described, and then through projection mechanisms. The angst experienced by the new mother is attributed to elements outside of the Self, such as a son or partner, which become “persecutory objects.” In this situation, this kind of personality organization is more likely to develop depression with prominent features of anxiety and dysphoria.

Our findings agree with data from previous research concerning the personality organization as a factor of vulnerability in perinatal depression, which had found two different premorbid personalities at the basis of perinatal depression, using different assessment tools. Some research has identified a specific bond between obsessive-compulsive or dependent personality traits and depressive perinatal risk [10, 12–14]. Vulnerability elements were linked to feelings of guilt and inadequacy, low self-esteem, and scarce autonomy, in association with rumination, lack of assertiveness, and hypersensitivity toward refusal. Alternatively, other studies have focused on a specific relation between BDP and perinatal depression, pointing out that mothers with borderline personality had difficulties in understanding the baby's moods and perceived themselves as incompetent parents. They experienced feelings of unfamiliarity, oppression, and anger while declaring themselves less satisfied with their maternity experience [16].

Finally, the present study identified a subgroup of women tested positive on the EPDS and did not show any kind of code type. This group had a personality profile that was quite normal (cluster 2). We labeled this group* “the defensive type”* as they did not portray any psychological disturbance on the MMPI-2 profile but seemed to have adopted a defensive disposition in answering questions.

In this group, the clinical scales were all in the standard range as well as almost all the content scales. The latter scales differ from the clinical scales because they are composed of all explicit items. In basic clinical scales, the subject may not be aware which indicators could be raised based on her answers; the scale content has an absolutely clear meaning. It seems rather strange, but also interesting, that women found to be positive on EPDS did not have any elevation on the scales of MMPI-2 related to depression. The 10 items that compose the EPDS explicitly refer to moments of sadness and unhappiness, fear for the future, and difficulties in sleeping and dealing with situations of any kind which is the same content of scale D and DEP (the Content Scale for Depression) of MMPI-2.

One possible explanation may lie in defensive attitude adopted by women of this group in answering to the questionnaire. Indeed, the control and defense scales appeared to be arranged in the typical V configuration of the subjects who were defensive in answering (L and K higher than F), tried to deny ongoing issues, or presented themselves in an ameliorative light. The F scale (“infrequency” = simulation), an indicator of psychopathological suffering, was very low compared with the other subgroups but the inclination of the thymic axis was oriented in the direction of depression. This configuration is often associated with people who present features of unexpressed anger, with significant elements of repression of their aggressive impulses. They try to avoid or deny unacceptable feelings, impulses, and problems, while trying to present themselves in the best way possible. As known from literature, one of the main reasons of this defensive attitude could be the fear of stigma, not just of mental illness but of being labelled a “bad mother” [40].

Moreover, these women seem to present a high level of psychopathology in clinical evaluation, in contrast to the psychometric results. They have areas of psychotic functioning, particularly with regard to the affective area, but they maintain good work functioning. We suppose that projective tests could be useful for the emergence of psychopathological elements clinically identified in this group, which are probably related to defense mechanisms that could not be detected by the measures used. Hence, based on these results, we must warn psychologists, psychiatrists, and other professionals about generalizing observations of the personality structure based on clinical experience.

Lastly, the three groups did not differ with regard to sociodemographic aspects, complications in pregnancy, and exposure to stressful life events. This result supports the hypothesis that personality structure is an important factor in determining how motherhood is experienced and how women could react to distress that can be experienced in this period of physical, psychological, and relational transformations. Moreover, personality profile could influence the clinical presentation of depressive symptoms, so it represents the vulnerability mechanism with which the pathology itself is established.

## 4. Conclusion

Based on these results, it can be assumed that severe personality structures can develop depressive manifestations during the delicate phase of transition to motherhood, in the presence of difficulties concerning the area of the integration of identity and the affective modulation. For these women, motherhood is not a phase of development that leads them to a more mature and integrated identity. Motherhood could represent a sort of “threat” because it exposes them to developmental challenges to find a new balance that they fail to achieve, falling into a depressive condition.

In line with literature data, we identified two principal personality profiles, in our sample. These profiles were associated with specific psychopathological features: (a) structures of personality with prevalent obsessive or dependent functioning, with tendencies to develop psychasthenic depression; (b) structures of personality with prevalent borderline functioning, with tendencies to develop dysphoric depression.

The presence of an “apparently normal” personality profile in our sample was probably due to the tendency in this group of patients to adapt to normative patterns and to adopt a defensive attitude (as the presence of V configuration in the control scales suggests), for fear of the stigma still present towards mental illness or of being labelled a “bad mother.” Since the psychometric results do not match with the clinical observation of psychopathological features, another likely cause could be the presence of defense mechanisms, such as psychotic denial or dissociation that cannot be detected by the measure used. Therefore, the use of projective tests (such as Rorschach test) might be useful for clarifying the characteristics of this group of patients.

In conclusion, despite perinatal depression falling within the framework of mood disorders, our work has enabled us to highlight how the characteristics of personality and interpersonal functioning have a key role in both the onset and the clinical manifestations of the disease.

As known from classical psychopathology, an event of particular existential value (the so-called “key event,” according to Kretschmer) can specifically affect areas of personality that are particularly fragile and have a specific pathogenic value. The reaction to such an event will assume different phenomenological configurations influenced by personality structure. Therefore, classifying personality profiles in women with perinatal depression appears to be fundamental for the following:

- implementing screening protocols with added questions about personality in order to promote early detection and intervention on at-risk populations (primary prevention);
- planning targeted therapeutic intervention, both from pharmacological and psychotherapeutic points of view, with focus on areas of personality that are particularly fragile and in relation to interactive and representational patterns (secondary prevention);
- providing clinicians engaged at different levels with the care of pregnant women and their children (obstetricians, gynecologists, neonatologists, and pediatricians) with instruments for understanding and timely diagnosis of at-risk psychopathological phenomena, in order to create a network with specialized centers to support the development of both motherhood and parenting skills.

This study had several limitations. First, the size of the sample was low, making the size of the groups derived from the cluster analysis also low; since a control group of pregnant nondepressed mothers was not used, the study population was compared with the population norm. Therefore, the investigation on a larger sample and a control group will be needed to make the results generalizable, especially with regard to group 3. Secondly, the personality of the individual is so multifaceted that many aspects will not be explained, even by complex tests such as MMPI-2. Although this test essentially highlights stable characteristics, we need to consider that the elevation of some scales could be influenced, at least in part, by the acute psychopathological state (as with scale D, e.g.). In this light, in the event that the depressive state is resolved, in the future, it could be useful to retest these women with MMPI-2 to differentiate which features are influenced by the acute psychopathological state and to confirm which ones are trait characteristics, even if we expect that these results are not to give substantial differences, based on the literature. Third, we mostly evaluated women's mental states with self-administered questionnaires. However, we can add that all data were corroborated by clinical observations and also by using SCID-I. Fourth, the second group represents the most difficult one to interpret because the psychometric characteristics do not match clinical evaluations. In this group, we found a V configuration of the validity scales, which has traditionally been an indicator of a defensive attitude. This could be linked to the presence of defense mechanisms or to the fear of stigma. Therefore, the use of other assessment tools, such as projective tests, could be recommended in future studies to clarify the characteristics of this group of patients, with regard to the possible presence of defense mechanisms that were not recognized by the psychometric instruments used in this research. Finally, it could be interesting to plan further studies that take into account the characteristics of personality in relation to attachment patterns. This would improve the understanding of the individual and interpersonal functioning of women who develop perinatal depression.

## Figures and Tables

**Table 1:** Characteristics of the study population.

Variable	Category	*N*	%
Civil status	Single	3	5%
	Married/cohabitant	47	85%
	Divorced	2	4%

Education	Primary school	6	11%
	High-school	27	49%
	College	19	35%

Job status	Student/housewive	7	13%
	Self-employed	6	11%
	Employee	27	49%
	Worker	7	13%
	Other	5	9%

Past pregnancies	No	29	53%
	One or more	21	38%

Obstetric complications	No	33	60%
	Yes	18	33%

Medical conditions	No	44	80%
	Yes	7	13%

Smokers	No	36	65%
	Yes	15	27%

Psychiatric history	No disorders	21	38%
	Mood disorder	4	7%
	Mood disorder, anxiety	7	13%
	Mood disorder, eating disorder	6	11%
	Anxiety	9	16%
	Eating disorder	8	15%

Previous perinatal disorders	No	46	84%
	Yes	6	11%

Familiar Psychiatric History	No	13	24%
	Yes	39	71%

Mourning	No	26	47%
	Yes	26	47%

Illness of a family member	No	32	58%
	Yes	23	42%

Job loss	No	41	75%
	Yes	11	20%

Economic difficulties	No	34	62%
	Yes	18	33%

Marital conflicts	No	28	51%
	Yes	24	44%

Family conflicts	No	39	71%
	Yes	13	24%

Note: due to occasional missing values the percentages do not sum up to 100%.

**Table 2:** MMPI-2 clinical scales: mean *T*-scores and standard deviations of three groups compared.

		Psychasthenic type	Defensive type	Dysphoric type
		M (SD)	M (SD)	M (SD)
L	Lie	54.88 (7.25)	52.10 (8.61)	46.00 (5.66)
F	Frequency	56.65 (8.38)	47.45 (7.29)	**74.33** (9.97)
K	Correction	47.59 (7.46)	48.10 (12.37)	42.33 (7.23)
HS	Hypochondria	61.12 (6.67)	53.80 (6.10)	**80.17** (10.34)
D	Depression	**74.12** (7.39)	51.85 (8.51)	**72.67** (10.93)
HY	Hysteria	61.53 (7.11)	53.70 (7.42)	**77.17** (9.75)
PD	Psychopathic deviation	63.12 (7.12)	54.95 (5.84)	**70.67** (7.84)
MF	Masculine-feminine	43.12 (7.19)	52.20 (10.33)	49.83 (8.64)
PA	Paranoia	57.41 (6.37)	51.15 (10.34)	**75.17** (7.08)
PT	Psychasthenia	**67.65** (6.52)	47.80 (6.01)	**75.67** (10.73)
SC	Schizophrenia	60.88 (5.21)	50.50 (6.69)	**76.17** (10.91)
MA	Mania	49.65 (9.19)	49.20 (7.61)	**65.83** (8.28)
SI	Social introversion	63.29 (8.75)	44.55 (7.91)	58.33 (5.32)

Note: *T*-scores were reported in bold if they were greater than 65.

**Table 3:** MMPI-2 content scales: mean *T*-scores and standard deviations of three groups compared.

		Psychasthenic type	Defensive type	Dysphoric type	*F*	Sig.
		M (SD)	M (SD)	M (SD)		
ANX	Anxiety	**66.24** (2.60)^a^	56.56 (2.68)^b^	**69.67** (4.37)^a^	4.83	∗∗
FRS	Fears	53.41 (2.40)^a^	52.19 (2.48)^a^	63.83 (4.05)^b^	3.20	∗
OBS	Obsessiveness	59.53 (1.89)^a^	47.81 (1.94)^b^	59.33 (3.17)^a^	10.63	∗∗
DEP	Depression	61.94 (2.13)^a^	50.69 (2.19)^b^	61.67 (3.58)^a^	7.66	∗∗
HEA	Health concern	56.88 (2.21)^a^	55.63 (2.28)^a^	**73.17** (3.72)^b^	8.81	∗∗
BIZ	Bizarre mentation	49.94 (2.06)^a^	50.06 (2.12)^a^	**60.50** (3.47)^b^	3.88	∗
ANG	Anger	51.41 (2.47)^a^	55.06 (2.54)^a^	**63.67** (4.15)^b^	3.23	∗
CYN	Cynicism	52.94 (2.27)	54.38 (2.34)	51.67 (3.82)	0.21	ns
ASP	Antisocial practices	49.82 (2.83)	50.38 (2.91)	50.83 (4.76)	0.02	ns
TPA	Type A	50.47 (2.13)	53.19 (2.20)	53.00 (3.59)	0.44	ns
LSE	Low self-esteem	59.12 (2.22)^a^	47.19 (2.29)^b^	60.33 (3.74)^a^	8.48	∗∗
SOD	Social discomfort	60.65 (2.31)^a^	43.31 (2.39)^b^	52.83 (3.90)^a^	13.62	∗∗
FAM	Family problems	55.12 (2.49)	51.31 (2.56)	58.67 (4.19)	1.27	ns
WRK	Work interference	63.41 (1.95)^a^	49.13 (2.01)^b^	**65.17** (3.29)^a^	15.92	∗∗
TRT	Negative treatment indicators	57.94 (2.12)^a^	49.69 (2.19)^b^	61.17 (3.57)^a^	5.41	∗∗

Note: ns indicated a not significant *F*-test. ∗ indicated a statistically significant *F*-test (*P* < .05). ∗∗ indicated a statistically significant *F*-test (*P* < .01). Duncan post hoc tests were carried out where appropriate. The same letters in superscript following values indicated no significant between-group difference. Different letters indicated statistical significance (*P* < .05).

**Table 4:** EPDS: mean scores and standard deviations of three groups compared.

	Psychasthenic type	Defensive type	Dysphoric type	*F*	Sig.
	M (SD)	M (SD)	M (SD)		
EPDS	19.41 (4.05)^a^	15.05 (3.91)^b^	24.00 (3.74)^c^	13.61	∗∗

Note: ∗∗ indicated a statistically significant *F*-test (*P* < .01). Different letters in superscript following values indicated a statistically significant Duncan post hoc test (*P* < .05).
